# DHA and therapeutic hypothermia in a short-term follow-up piglet model of hypoxia-ischemia: Effects on H+MRS biomarkers

**DOI:** 10.1371/journal.pone.0201895

**Published:** 2018-08-07

**Authors:** Marianne Ullestad Huun, Håvard Garberg, Else Marit Løberg, Javier Escobar, Jose Martinez-Orgado, Ola Didrik Saugstad, Rønnaug Solberg

**Affiliations:** 1 Department of Pediatric Research, Women and Children's Division and Institute for Surgical Research, Oslo University Hospital, Rikshospitalet, Oslo, Norway; 2 University of Oslo, Oslo, Norway; 3 Department of Pathology, Oslo University Hospital, Ullevål, Oslo, Norway; 4 Neonatal Research Unit, Health Research Institute Hospital La Fe, Valencia, Spain; 5 Division of Neonatology, Hospital Clinico San Carlos-IdISSC, Madrid, Spain; 6 Department of Pediatrics, Vestfold Hospital Trust, Tønsberg, Norway; Loma Linda University School of Medicine, UNITED STATES

## Abstract

**Background:**

Therapeutic hypothermia has become the standard of care for newborns with hypoxic-ischemic encephalopathy in high and middle income countries. Docosahexaenoic acid (DHA) has neuroprotective properties of reducing excitotoxicity, neuroinflammation and apoptosis in rodent models. We aim to study whether post hypoxic administration of i.v. DHA will reduce H^+^MRS biomarkers and gene expression of inflammation and apoptosis both with and without hypothermia in a large animal model.

**Methods:**

Fifty-five piglets were randomized to severe global hypoxia (N = 48) or not (Sham, N = 7). Hypoxic piglets were further randomized by factorial design: Vehicle (VEH), DHA, VEH + Hypothermia (HT), or DHA + HT. 5 mg/kg DHA was given intravenously 210 min after end of hypoxia. Two-way ANOVA analyses were performed with DHA and hypothermia as main effects.

**Results:**

Cortical lactate/N-acetylaspartate (Lac/NAA) was significantly reduced in DHA + HT compared to HT. DHA had significant main effects on increasing N-acetylaspartate and glutathione in hippocampus. Therapeutic hypothermia significantly reduced the Lac/NAA ratio and protein expression of IL-1β and TNFα in hippocampus and reduced Troponin T in serum. Neuropathology showed significant differences between sham and hypoxia, but no differences between intervention groups.

**Conclusion:**

DHA and therapeutic hypothermia significantly improve specific H^+^MRS biomarkers in this short-term follow up model of hypoxia-ischemia. Longer recovery periods are needed to evaluate whether DHA can offer translational neuroprotection.

## Introduction

Perinatal asphyxia is a major cause of infant and pediatric disability projecting into adulthood. Therapeutic hypothermia is the only established treatment for moderate to severe encephalopathy. However, there is need for adjuvant strategies.

Docosahexaeonic acid (DHA) is an omega-3 fatty acid highly concentrated in the developing brain [1]. It is a constituent of neuronal membranes in grey matter, white matter and also in glial cells. DHA has antioxidant properties and acts as a therapeutic agent. It has been shown to reduce inflammation, excitotoxicity and prevent brain volume loss in animal models of stroke [2–4]. Post-insult injected and dietary DHA has elicited neuroprotection in experimental animal models of traumatic brain injury, spinal cord injury and white matter damage [5, 6]. In a newborn rat model DHA augmented the beneficial effect of hypothermia on reducing brain volume loss and also improved the rats’ behavioral motor pattern [7]. The effects of DHA and therapeutic hypothermia have to our knowledge not been investigated in a large animal model.

Proton magnetic resonance spectroscopy (H^+^MRS) is often performed after the infant is rewarmed and weaned from the ventilator. Currently, H^+^MRS and amplitude integrated EEG are considered the best predictors for neurodevelopmental outcome at 18 months following perinatal encephalopathy [8, 9]. The H^+^MRS biomarker N-acetyl aspartate (NAA) to lactate (Lac) ratio (Lac/NAA) is the most accurate quantitative MRS biomarker within the neonatal period (1–30 days) for prediction of neurodevelopmental outcome after neonatal encephalopathy and is recommended as a surrogate endpoint in trials evaluating novel neuroprotective strategies[10]. H^+^MRS on intact frozen tissue is considered a good surrogate for in vivo imaging [11].

Previously, we have shown how DHA reduces lipid peroxidation in urine and brain tissue of hypoxic-ischemic piglets [12, 13]. In the present study we aim to measure how post-insult administration of DHA will affect brain H^+^MRS biomarkers in piglets treated with and without therapeutic hypothermia, contributing to the existing knowledge gap in translational medicine of how these treatments effect a large animal model. As the current experiment is part of a larger study design, we also evaluate neuropathology, inflammatory protein levels in two sections of the brain, S100b in CSF and Troponin T in serum as markers of hypoxic injury hypothesizing reduced levels of neuroinflammation and glial and myocardial biomarkers in piglets treated with therapeutic hypothermia or DHA or both.

## Methods

### Approval

The Norwegian Council for Animal Research approved the experimental protocol (No 5723). The animals were cared for and handled in accordance with the European Guidelines for Use of Experimental Animals, by certified FELASA (Federation of European Laboratory Animals Science Associations) Category C researchers.

### Experimental design

The current study is part of a larger experimental design of eighty-one newborn Noroc (LyxLD) pigs randomized to interventions of either DHA, cannabidiol (CBD) or therapeutic hypothermia (S1 Fig). The two studies have three shared groups (VEH, Hypothermia and Sham) in order to limit the number of animals needed. The CBD part of the study has been published [14]. The piglets had inclusion criteria of 12–36 h of age, Hb >5 g/dL and good general condition.

**Fig 1:**
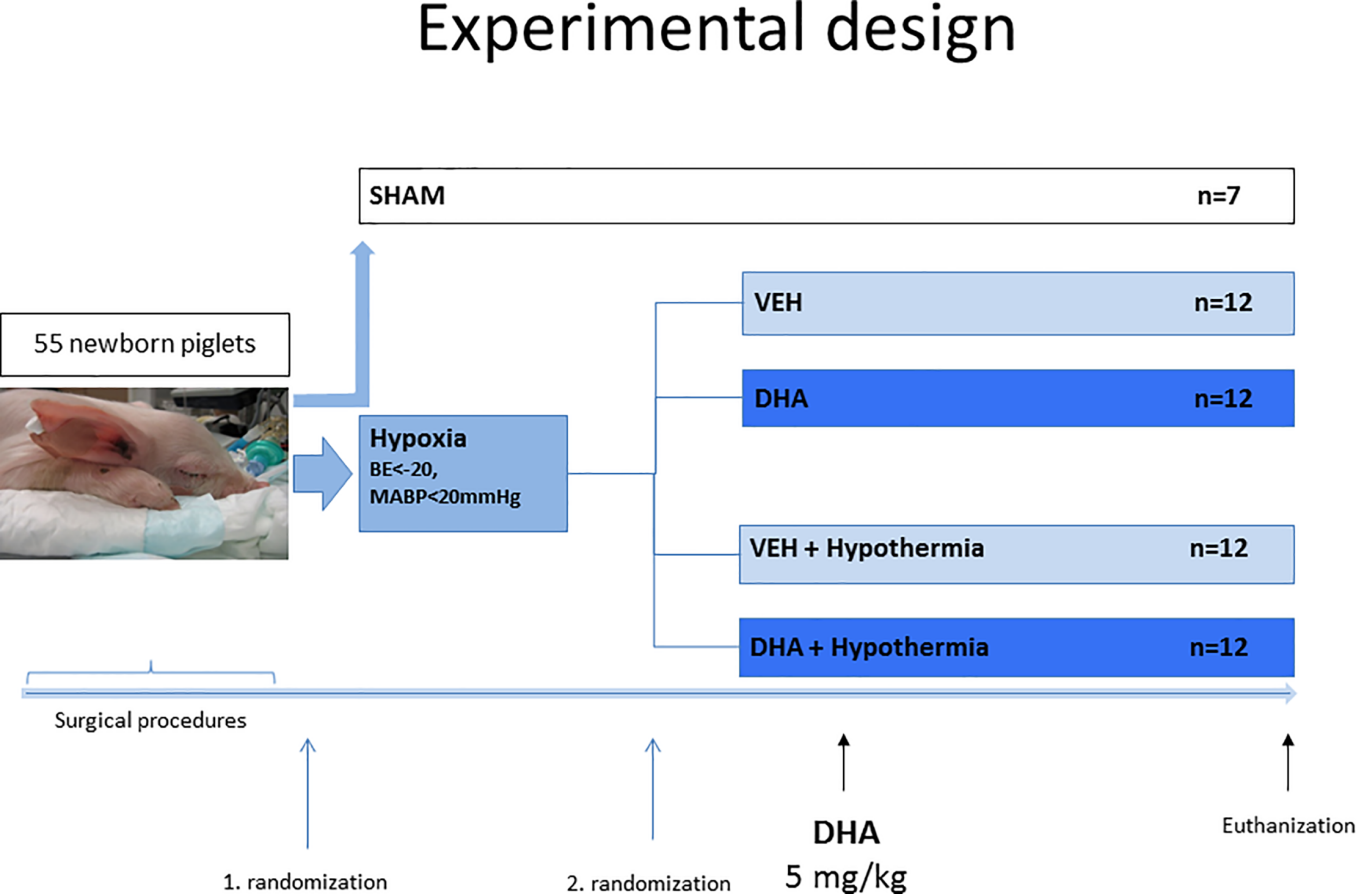
Experimental design. Fifty-five piglets underwent anesthesia and instrumentation for continuous monitoring of blood pressure, saturation, heart rate and rectal temperature. 48 piglets were subjected to hypoxia-ischemia (HI) by 8% O_2_ on the endotracheal tube until severe acidosis (arterial base excess (BE) -20 mmol/l) and/or hypotension (mean arterial blood pressure <20 mm Hg). After reoxygenation with room air, piglets were randomized to one out of four intervention groups by factorial design: i) Vehicle (VEH) (N = 12), ii) DHA (N = 12), iii) VEH + Hypothermia (HT) (N = 12) and iiii) DHA + HT (N = 12). Hypothermia was started immediately after this second randomization. DHA was given 210 minutes after the end of hypoxia and piglets were euthanized 9.5 hours after the end of hypoxia.

Forty-eight of fifty-five piglets were subjected to experimental interventions and block randomized twice by factorial design (Fig 1). A sham group of seven piglets underwent sham operation but did not receive hypoxia. The methods are previously described in detail [12]. Briefly, piglets were anesthetized and intubated and hypoxia induced by 8% O_2_ on the endotracheal tube. When base excess reached -20 or mean arterial blood pressure <20 mmHg the piglets were resuscitated with 21% O_2_. Throughout the experiment temperature-corrected arterial blood gasses were collected at set timepoints.

Piglets randomized to therapeutic hypothermia were immediately cooled with a cooling mattress (Tecotherms TSmed 200; TecCo, Halle, Germany) with target rectal temperature 34.5°C. DHA (1 g CIS-4, 7, 10, 13, 16, 19- docosahexaenoic acid) was dissolved in 0.9% NaCl and diluted to 10 mg/ml. Piglets randomized to DHA received 5 mg/kg intravenously 3.5 hours after end of hypoxia. Following our established model piglets were euthanized 9.5 hours after end of hypoxia with 150 mg/kg intravenous pentobarbital. The piglets were cooled on plates of dry ice while the autopsy was performed. Tissues from prefrontal cortex and hippocampus were immediately excised and snap frozen in liquid nitrogen and stored at -80°C.

### Proton-magnetic-resonance-spectroscopy (H^+^MRS)

The details of the H+MRS have been previously described [14]. Briefly, H+MRS was performed in the MRI Unit of the Instituto Pluridisciplinar (Universidad Complutense, Madrid, Spain) at 500.13 MHz using a Bruker AMX500 spectrometer 11.7 T operating at 4°C on frozen brain samples from hippocampus and prefrontal cortex (5–10 mg weight). Standard solvent suppressed spectra were acquired into 16 k data points, averaged over 256 acquisitions, total acquisition ∼14 min using a sequence based on the first increment of the NOESY. A spectral width of 8,333.33 Hz was used. All spectra were processed using TOPSPIN software, version 1.3 (Bruker, Rheinstetten, Germany). Curve fitting was performed by using the 3.1.7.0 version of the SpinWorks software (University of Manitoba, Winnipeg, Canada), and concentrations and ratios were calculated, including: N-acetylaspartate (NAA), lactate/N-acetylaspartate (Lac/NAA), glutamate/N-acetylaspartate (Glu/NAA) ratios and glutathione (GSH).

### Biomarkers

S100 calcium-binding protein B (S100B) in CSF and plasma Troponin-T were measured on commercially available immunoassay kits (Elecsys Troponin T high sensitive and Elecsys S100, Roche Diagnostics, Mannheim, Germany) using an electrochemie luminescence immunometric assay (ECLIA) on the Cobas e601 immunoassay platform. Troponin T is detected at a limit of 5 ng/L and with a 10% coefficient of variation precision of 13 ng/L. S100b is detected at a limit of 0.005 μg/L with a precision of 0.7–1.8% and reproducibility of 2.5–3.1% according to the manufacturer.

### Protein expression

We selected cytokines IL-1β, IL-6 and TNFα as markers of inflammation. 100 mg of cerebral tissues from prefrontal cortex and hippocampus were homogenized in ice-cold lysis buffer (TrisHCl (pH 7.5) with 1% NP-40 and a protease inhibitor cocktail (Sigma-Aldrich, St. Louis, MO). Protein concentration was measured by the BCA method (Pierce, Cheshire, UK). Cytokines were analyzed by a commercially available enzyme immunoassay, performed according to the manufacturer´s instructions (R&D Systems, Oxford, UK).

### Pathology

Brain extraction and preparation methods are previously described [14]. Neuropathological analysis of H&E brain sections was done by a pathologist widely experienced with evaluating hypoxic-ischemic piglet brains. The pathologist was blinded to the randomization and the clinical outcome. A modified version of a validated scoring system was used [15]. Six regions (prefrontal, parietal and dorsal cortex, hippocampus, white matter and cerebellum) were analyzed and summarized to a total score ranging from 0 to 4. Zero representing no damage, 1: mild/moderate, 2: moderate/severe, 3: severe, and 4: massive damage with autolysis of the cerebrum.

### Statistics

Statistics were done using GraphPad Prism 6 (GraphPad Software Inc., San Diego, CA, USA) and SPSS 23 (SPSS, Chicago, IL). Shapiro-Wilk’s test was applied to test for normality on the data and log-transformation was performed on non-normally distributed data. H+MRS biomarkers were tested in a two-way ANOVA analysis with DHA and hypothermia as main factors, meaning groups treated with DHA were DHA and DHA + HT and groups treated with hypothermia were VEH + HT and DHA + HT. The reciprocal groups were non-DHA (VEH and HT) and non-HT (VEH and DHA). Sham piglets are not included in the main model. First, we tested for interaction between the two main factors, if p≤0.10 post hoc two-by-two group comparisons were further made with one-way ANOVA. P-values were adjusted by Fisher’s test. However, if interactions were not present only main effects were maintained in the model. P-values <0.05 were considered statistically significant for both one-way and two-way ANOVA analysis. All figures are displayed with mean ± SD.

## Results

### Physiology

There were no differences in postnatal age, body weight or baseline physiology between the randomized groups (Table 1). Nor were there any differences in biomarker results between males and females. One piglet in the DHA group was excluded due to massive autolysis upon autopsy. The study had a mortality rate of 8% related to cardiac arrest during or shortly after hypoxia-ischemia.

**Table 1:** Physiology of the intervention groups throughout the experiment.

Measurements	VEH	DHA	VEH + HT	DHA + HT	Sham	p-value
**Body weight (g)**	1970 (100)	2012 (136)	2006 (118)	2055 (97)	2028 (24)	0.43
**Sex (male:female)**	1.4	1.4	0.5	0.5	0.75	0.55
**Post-natal age (h)**	25.7 (2.6)	26.7 (5.1)	26.8 (3.3)	25.3 (4.2)	25.3 (2.8)	0.79
**Hypoxia time (min)**	41.5 (10)	42.1 (18)	41.4 (10)	41.4 (17)	0.0	1.0
**Cooling time (min)**	0	0	465	474	0	**0.00**
**Hemoglobin (g/dl)**	7.5 (1.1)	7.3 (1.4)	7.8 (1.4)	7.3 (1.3)	7.8 (1.0)	0.87
**Base Excess (mmol/L)**						
Start Hypoxia	1.7 (2.3)	2.0 (3.0)	1.3(3.4)	2.5 (3.0)	0.5 (2.7)	0.65
End Hypoxia	-20.2 (1.2)	-20.3 (0.4)	-20.0 (0.5)	-19.5 (3.2)	0.3 (1.4)	**0.00**
3.5 h	0.6 (2.5)	-0.2 (2.7)	-0.1 (2.5)	0.5 (2.2)	1.9 (2.4)	**0.017**
End study	1.7(2.9)	-1.1 (3.5)	-2.8 (3.8)	0.3 (3.0)	-0.3 (3.5)	**0.046**
**Lactate (mmol/L)**						
Start Hypoxia	2.2 (0.8)	2.025 (0.6)	1.9 (0.6)	2.0 (0.8)	2.4 (1.0)	0.70
End Hypoxia	15.7 (2.5)	16.9 (2.9)	16.2 (1.9)	15.7 (2.3)	1.7 (0.4)	**0.00**
3.5 h	1.9 (0.8)	2.0 (1.6)	1.8 (0.7)	2.0 (1.5)	1.4 (0.3)	0.78
End study	1.3 (0.4)	1.5 (2.0)	2.0 (1.6)	1.3 (0.4)	1.1 (0.3)	0.48
**Temperature (°C)**						
Start Hypoxia	39.3 (0.7)	39.3 (0.7)	39.3 (0.5)	39.4 (0.8)	39.6 (0.6)	0.90
End Hypoxia	38.6 (0.5)	38.4 (0.5)	38.2 (0.5)	38.5 (0.3)	39.5 (0.8)	**0.000**
3.5 h	39.5 (0.7)	39.0 (0.6)	35.1 (0.4)	35.2 (0.7)	39.6 (0.7)	**0.000**
End study	39.2 (0.6)	38.9 (0.8)	35.0 (0.5)	35.1 (0.4)	39.1 (0.5)	**0.000**
**Mean arterial blood pressure (mmHg)**						
Start Hypoxia	65.4 (11.4)	62.6 (11.5)	68.5 (7.5)	60.5 (9.7)	69.3 (10.8)	0.33
End Hypoxia	43.1 (18.4)	47.1 (21.4)	39.6 (18.4)	48.3 (24.7)	71.4 (13.9)	**0.017**
3.5 h	58.3 (10.9)	54.5 (9.1)	61.9 (10.4)	53.4 (10.5)	59.6 (7.5)	0.44
End study	56.3 (7.5)	51.3 (13.1)	49.9 (9.7)	51.6 (13.0)	56.1 (3.6)	0.49
**pO2 (kPa)**						
Start Hypoxia	9.3 (0.22)	9.77 (0.56)	9.36 (0.68)	10.29 (0.37)	9.65 (0.40)	0.59
End Hypoxia	4.69 (0.99)	4.32 (0.5)	4.67 (0.5)	4.46 (0.59)	10.6 (1.3)	**0.001**
3.5 h	9.9 (1.0)	10.3 (1.9)	8.4 (0.86)	8.9 (1.4)	10.4 (1.2)	**0.003**
End study	9.9 (1.1)	9.4 (1.3)	9.4 (1.5)	9.8 (1.9)	10.5 (1.9)	0.51
**pCO2 (kPa)**						
Start Hypoxia	5.14 (0.71)	5.7 (2.0)	5.6 (0.6)	5.2 (0.6)	5.6 (0.6)	0.59
End Hypoxia	8.8 (1.4)	9.3 (1.3)	9.2 (0.8)	9.2 (1.3)	5.5 (1.9)	**0.000**
3.5 h	5.0 (0.8)	4.3 (0.7)	5.7 (0.7)	4.7 (0.5)	4.8 (0.7)	**0.000**
End study	4.5 (0.6)	4.9 (0.6)	5.1 (0.9)	4.5 (0.6)	4.7 (0.5)	0.088
**Glucose (mmol/L)**						
Start Hypoxia	7.0 (1.4)	7.4 (1.1)	7.5 (1.8)	6.7 (1.6)	7.5 (0.9)	0.61
End Hypoxia	11.1 (5.3)	10.3 (3.5)	11.5 (5.0)	10.2 (5.8)	6.6 (1.1)	0.25
3.5 h	5.7 (1.6)	5.5 (1.1)	5.5 (0.9)	5.5 (2.3)	5.7 (0.6)	0.99
End study	5.1 (1.0)	5.5 (0.9)	5.6 (1.1)	5.3 (1.3)	5.3 (0.7)	0.86
**pH**						
Start Hypoxia	7.44 (0.05)	7.44 (0.06)	7.4 (0.06)	7.44 (0.06)	7.4 (0.06)	0.11
End Hypoxia	6.86 (0.05)	6.84 (0.04)	6.85 (0.03)	6.86 (0.08)	7.43 (0.04)	**0.00**
3.5 h	7.43 (0.07)	7.47 (0.08)	7.38 (0.06)	7.45 (0.06)	7.47 (0.08)	**0.017**
End study	7.43 (0.07)	7.41 (0.08)	7.38 (0.09)	7.46 (0.06)	7.44 (0.04)	**0.046**
**HR (bpm)**						
Start Hypoxia	177 (52)	168 (30)	153 (28)	163 (28)	152 (35)	0.50
End Hypoxia	190 (34)	173 (25)	185 (52)	188 (27)	158 (40)	0.35
3.5 h	214 (50)	208 (66)	163 (29)	178 (37)	162 (31)	**0.027**
End study	197 (53)	208 (53)	144 (30)	170 (46)	168 (42)	**0.019**

Values listed as mean (±SD). Significant group differences (one-way ANOVA): p-values in bold typing. The groups were comparable as there were no intergroup differences in body weight, post-natal age, hypoxia time or baseline physiological (heart rate, mean arterial blood pressure) and biochemical measures (blood lactate, base excess and glucose). 3.5 h indicates hours after the end of hypoxia and at which time point the DHA-randomised piglets received DHA. End study is 9.5 hours after the end of hypoxia.

### Proton magnetic resonance spectroscopy (H^+^MRS)

Results of the main effects and mean values for the randomized groups are displayed in the S1 and S2 Tables.

#### Effect of DHA

**Fig 2:**
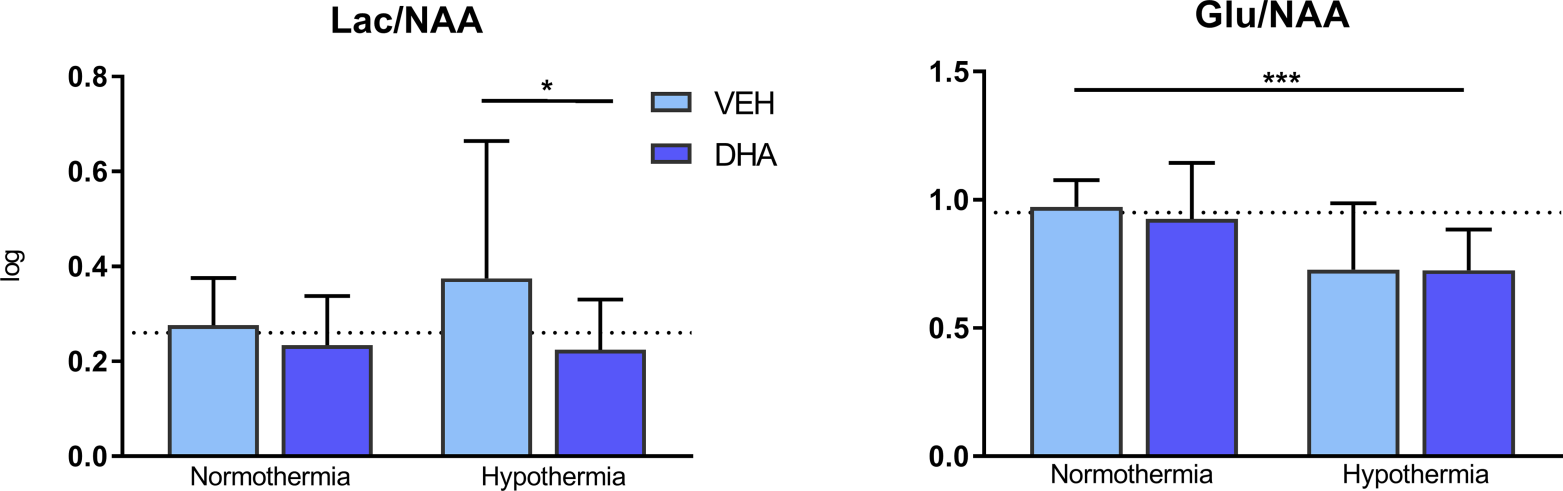
H^+^MRS biomarkers in prefrontal cortex. Lac/NAA was significantly reduced in the DHA + Hypothermia group compared to DHA + VEH. Hypothermia significantly reduced Glu/NAA compared to normothermia. p<0.05, *** p<0.001. Dotted line representing mean value in sham piglets.

In cortical tissue there was a weak interaction between DHA and HT on Lac/NAA, p = 0.081. Further one-way ANOVA analysis showed that Lac/NAA was reduced in the DHA + HT group compared to the VEH + HT group (0.22 ± 0.10 vs 0.37 ± 0.29 log ppm, p = 0.040) (Fig 2).

**Fig 3:**
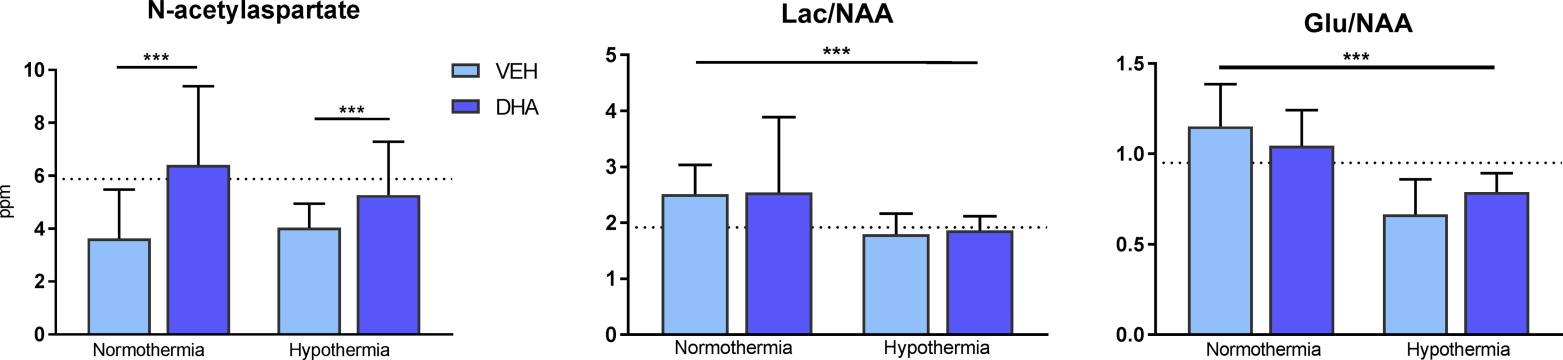
H^+^MRS biomarkers in hippocampus. N-acetylaspartate (NAA) was significantly increased in the DHA treated groups. Lac/NAA and Glu/NAA were significantly reduced in the hypothermia treated piglets. Dotted line representing mean value in sham piglets.

In hippocampal tissue there was a main effect of DHA on NAA (DHA 6.0 ± 2.4 vs Non-DHA 3.8 ± 1.5 ppm, p = 0.001) (Fig 3). DHA had a significant main effect on increasing GSH (0.41 ± 0.25 vs 0.23 ±0.18, p = 0.009) (S1 Table).

#### Effect of hypothermia

In cortical tissue (Fig 2), there was a main effect of HT on reducing Glu/NAA (mean ± SD: HT 0.72 ± 0.20 vs Non-HT 0.95 ± 0.16, p<0.0001).

In hippocampal tissue (Fig 3), there was a main effect of HT on decreasing Lac/NAA (1.83 ± 0.31 vs 2.35 ± 0.49, p<0.0001) and Glu/NAA (0.73 ± 0.16 vs 1.09 ± 0.22, p<0.0001). There was no effect of hypothermia on NAA.

### Biomarkers in CSF and blood

There was interaction between HT and DHA on S100b in CSF (p = 0.049). S100b was significantly higher in the HT than in the VEH treated group (17.2 ±19.2 vs 5.2 ± 2.2 ng/ml, p = 0.035).

**Fig 4:**
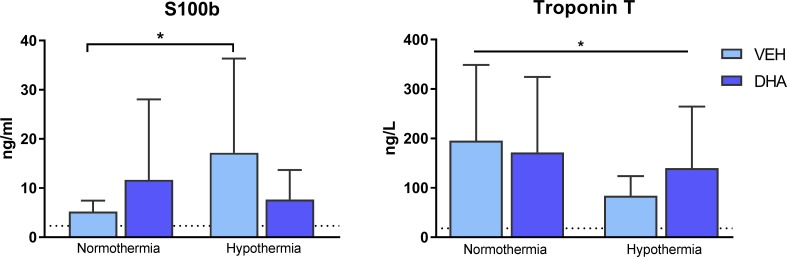
Biomarkers in CSF and blood. S100B in CSF was significantly increased in VEH + HT compared to VEH (p = 0.035). In serum, Troponin T was significantly decreased in groups treated with therapeutic hypothermia compared to normothermia (p = 0.018, two-way ANOVA). Dotted line representing mean value in sham piglets.

There was a significant main effect of HT on plasma Troponin-T (HT 95 ± 45 vs Non-HT 180 ± 152 ng/L, p = 0.018, Fig 4).

### Protein expression in cortex and hippocampus

**Fig 5:**
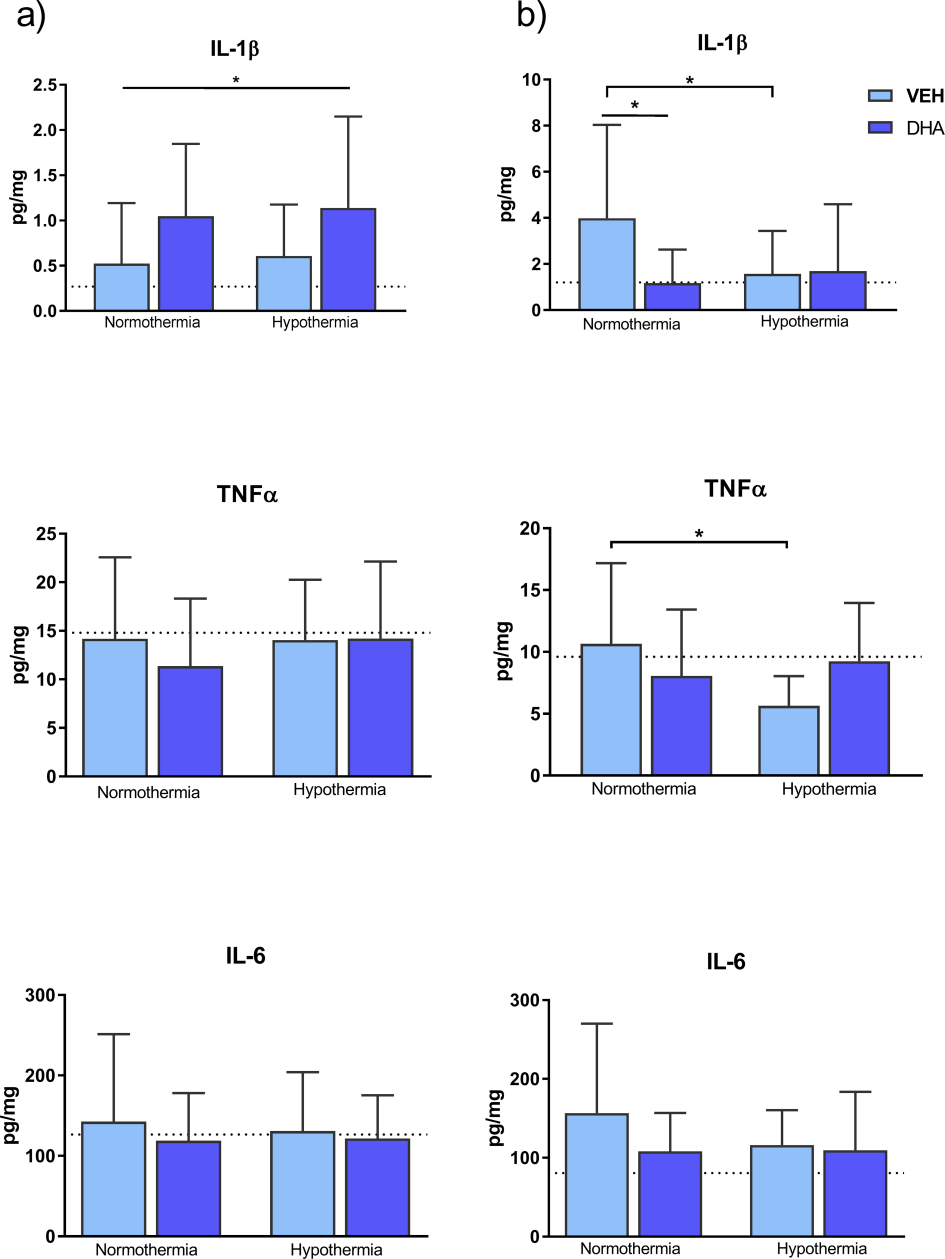
**Protein expression from a) prefrontal cortex and b) hippocampus**. In cortex, DHA significantly increased IL-1β. In hippocampus, IL-1β was significantly reduced in both DHA and VEH + HT compared to VEH. Dotted line representing mean value in sham piglets. * p<0.05.

In cortex we found a significantly increased main effect of DHA on IL-1β compared to Non-DHA (1.09 ± .89 vs 0.57 ± .60 pg/mg, p = 0.027, Fig 5). There were interactions present in hippocampus on IL-1β (p = 0.085) and TNFα (p = 0.046). One-way ANOVA showed IL-1β was significantly reduced in both DHA compared to VEH (1.1 ± 1.4 vs 3.9 ± 4.0 pg/mg, p = 0.021) and VEH + HT compared to VEH (1.6 ±1.8 vs. 3.9 ± 4.0, p = 0.047). TNFα was significantly reduced in VEH + HT compared to VEH (5.6 ± 2.4 vs. 10.7 ±.5, p = 0.023).

### Neuropathology

**Fig 6:**
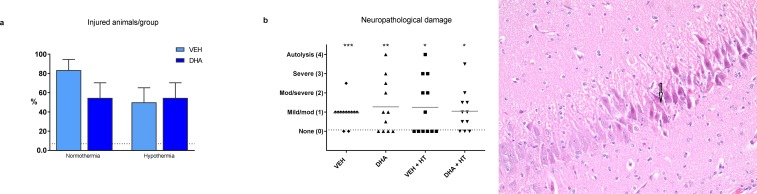
Neuropathology. Neuropathological analysis of brains from newborn piglets 9.5 h after hypoxia-ischemia treated with VEH, DHA, VEH + HT and DHA + HT. a) Percentage of animals with damaged cells (neuropathology score grade 1–4). b) Distribution of individual piglets on the neuropathology score scale. Data presented as individual scores (dots), grey lines representing group mean. Dotted line representing mean value in SHAM. c) Example of grade 2 damage of HE-staining from hippocampus. Arrow pointing at pycnotic neuron.

Fifty three piglet brains were subject to histopathology as one sample (DHA + HT group) was lost to follow up. Significant differences were found between all intervention groups and SHAM when comparing neuropathological damage (Grade 1–4) to no damage (Grade 0), (ANOVA, p = 0.009, Fig 6). Neuropathology score was significantly increased in VEH (mean, SD: 0.83 ± 0.39) compared to SHAM (0.14 ± 0.38, p = 0.002). There were no significant differences between the intervention groups.

## Discussion

To our knowledge, there are no large animal models which have treated hypoxia-ischemia with DHA and therapeutic hypothermia. Piglets are a favorable model in perinatal translational medicine due to size, degree of myelinisation and similar stage of brain development as newborn infants [16, 17]We wanted to investigate both main factors of DHA and hypothermia to look for positive interactions where DHA might have an augmentive effect on H^+^MRS biomarkers, but also to establish whether DHA could have neuroprotective potential alone as not all infants are born in facilities which can provide intensive care medicine required for therapeutic hypothermia. We found that DHA significantly increased NAA and GSH in deep grey matter, and cortical Lac/NAA was significantly reduced in the hypothermic DHA group compared to hypothermia alone. Therapeutic hypothermia reduced cortical and hippocampal ratios of Glu/NAA and Lac/NAA.

### Lac/NAA

We found a positive interaction between DHA and hypothermia in cortical Lac/NAA where DHA + HT reduced Lac/NAA more than HT alone (Fig 2). In a 2014 review by Robertson et al, deep grey matter Lac/NAA in encephalopatic newborns was concluded to be the most specific and sensitive spectroscopy biomarker to detect abnormal neurological outcome at 18 months [9]. Lac/NAA increases already at 2, 6 and 12 hours post asphyxia, where the earliest time point is connected to the immediate damage and later time points reflect the beginning of the secondary energy failure [18]. Significant reductions in brain excitotoxins have been shown 6 and 8 hours post-hypoxia in hypothermic piglets and increased Lac/NAA ratio 10 hours post-hypoxia [19, 20]. In piglets, Lac/NAA has also been shown to correlate with reduced apoptosis on immunohistochemistry after 48 hours [20]. Although a short follow-up time in the present study, the hypothermic piglets had significantly reduced Lac/NAA in both in prefrontal cortex and the hippocampus (Figs 2 and 3) in line with previous studies [14, 20, 21]. Lac/NAA was the only biomarker in which we found DHA to be significantly reduced compared to hypothermia (Fig 2). This novel finding may suggest that there could be a beneficial effect of DHA in addition to hypothermia treatment, but the results are not homogenous, meaning they have to be interpreted cautiously.

### NAA

We found that piglets treated with DHA maintained a significantly higher NAA concentration in hippocampal tissue. NAA is considered a neuronal marker due to its localization primarily in neurons, although there is a small presence in non-neuronal cell types like progenitor oligodendrocytes [22, 23]. Cheong et al published how MRS absolute quantitation of NAA concentration provided a more sensitive and specific biomarker of neurodevelopmental outcome in neonatal encephalopathy than Lac/NAA peak area ratio [24]. Deep grey matter NAA was the only MRS biomarker in this study to differentiate between control, mild and severe/fatal outcome during the first 3 days of life. NAA levels in the hippocampus are closely correlated to neuronal loss and were concluded to be a valuable marker of brain injury, given the ischemia was global and not focal [25]. In focal ischemia NAA was thought to be trapped in the dying neurons inside the penumbra and could thus be “falsely” elevated. Our model is a global hypoxia model and the sustained high levels of NAA in groups treated with DHA may be interpreted as diminished neuronal loss in deep grey matter indicative of neuroprotection. But the roles of NAA have not been fully understood as it potentially has a mulititude of roles including mitochondrial metabolism and the present results must thus be interpreted with caution [26].

### Glu/NAA

The immature brain expresses a high number of glutamate receptors and the effect of increased intracellular glutamate stores may be deleterious when there is insufficient ATP to maintain a low extracellular glutamate concentration by glutamate transporters. In this case, energy failure will lead to neuronal cell death. The Glu/NAA ratio has been found closely related to excitotoxic brain damage in infants, and hypothermia has been shown to reduce the Glu/NAA ratio already at 6 hours post hypoxia in newborn piglets [27, 28]. This is consistent with our findings of significantly reduced Glu/NAA in both cortex and hippocampus of hypothermic piglets (Figs 2 and 3). We did not find reductions by DHA.

### GSH

GSH is one of the most abundant anti-oxidants in the body and important in the highly oxidative brain. GSH protects the cell against reactive oxygen compounds and a GSH depletion will result in mitochondrial dysfunction and decreased NAA levels [29]. In the present study there were higher hippocampal levels of both NAA and GSH in the DHA treated animals, reflecting a more intact reductive capacity and maintained neuronal function compared to non-DHA treated animals.

### Inflammatory proteins in cortex and hippocampus

DHA reduced neuroinflammation after both traumatic brain injury and in models of stroke [2–4]. Chang et al showed how DHA reduced LPS induced inflammation in both neurons and microglia in cultured hippocampal brain cells [30]. We found regional effects of DHA on inflammation. In cortex, DHA increased IL-1β levels indicating increased inflammation (Fig 5). However, in the hippocampus both DHA and HT groups had significantly reduced levels of IL-1β compared to VEH as an expression of reduced inflammation. Hypothermia significantly reduced TNFα in the hippocampus compared to VEH. The newborn piglet brain can elicit regional differences in vulnerability as we have seen in one of our previous models on lipid peroxidation[13], which may help explain the unexpected increase of IL-1β in cortex, but yet it is an unexpected finding not supported by previous studies. The neuroinflammation following newborn hypoxia-ischemia is a multifaceted and complex process and work is still needed to fill the knowledge gap of how the various inflammatory molecules interact in the post-hypoxic phase [31].

### S100B and Troponin T

S100b is a neurotrophic factor secreted from astrocytes and is increasingly used clinically. Cord blood S100b was positively associated with both severity of encephalopathy and the risk of neurodevelopmental sequelae [32]. A time profile study of S100b in cooled infants showed that S100b was significantly decreased compared to normothermia at 48 hours post asphyxia, but not earlier at 6, 12 or 24 hours [33]. We found that therapeutic hypothermia significantly increased S100b in our study (Fig 4). We may speculate that this perhaps is due to the early sampling time point of 9.5 hours post asphyxia.

Therapeutic hypothermia significantly reduced Troponin T in the present study and this supports previous findings [34].

### Strengths and limitations

The model is supported by Domoki et al’s recommendations of utilizing hypoxic ventilation with systemic hypotension to reproduce the ischemic aspect of HIE in piglets which is superior to ischemic models of isolated carotid clamping [35]. The large animal piglet model is well suited for translational medicine. Ours is a newborn model of asphyxia as piglets have gone through perinatal transition. The dosage and timing of DHA administration are based on previous rat studies and it is not studied whether this is optimal in piglets, but delayed timing is clinically relevant.

Due to few piglets in each group and multiple comparisons, we chose to investigate further p-values ≤0.10 for the interaction level of the two-way ANOVA. This was to allow for more subtle interactions to be revealed, since they are more difficult to detect than main effects which the study was originally intended for. The alpha level for the main effects and for the one-way ANOVA is p<0.05.

Our model has a short follow-up time of 9.5 hours post end hypoxia limiting the possibility to assess neuroprotection by DHA. Histopathology will mainly reveal the immediate damage caused by the insult and thus verify the model. The model gives insight to processes occurring in the latent and early second-injury phase of the HIE development.

## Conclusion

Early protection by DHA is supported by biochemical improvement in NAA, GSH, and IL-1beta in deep grey matter. DHA in combination with hypothermia significantly reduced cortical Lac/NAA more than VEH + hypothermia, although this was not sustained in neuropathology at 9.5 hours after hypoxia. Longer recovery periods in a large animal model are needed to elucidate whether DHA can offer translational neuroprotection.

## Supporting information

S1 Fig Experimental design DHA + CBD. (PDF)

S1 Table Main effects of H^+^MRS biomarkers. Main effects of DHA and Hypothermia on H+MRS values shown as mean ppm ± SD. The two-way ANOVA gives the following groups: DHA (DHA pooled with DHA + HT), Non-DHA (VEH pooled with VEH + HT), HT (HT pooled with HT + DHA) and Non-HT (VEH + DHA). First column representing the interaction p-value between the main factors. Significant p-values in bold typing. * Interaction with p<0.10 with subsequent one-way ANOVA analysis and post-hoc Fisher’s test on the randomized goups (values in S2 Table). Non detectable (ND).
(DOCX)

S2 Table H^+^MRS biomarkers by randomized groups. (DOCX)
