# Supplementary figures and images for: DHA and therapeutic hypothermia in a short-term follow-up piglet model of hypoxia-ischemia: Effects on H+MRS biomarkers
Source: PLoS One. 2018 Aug 7;13(8):e0201895. doi: 10.1371/journal.pone.0201895 (PMC6080779; doi:10.1371/journal.pone.0201895)

# Experimental design

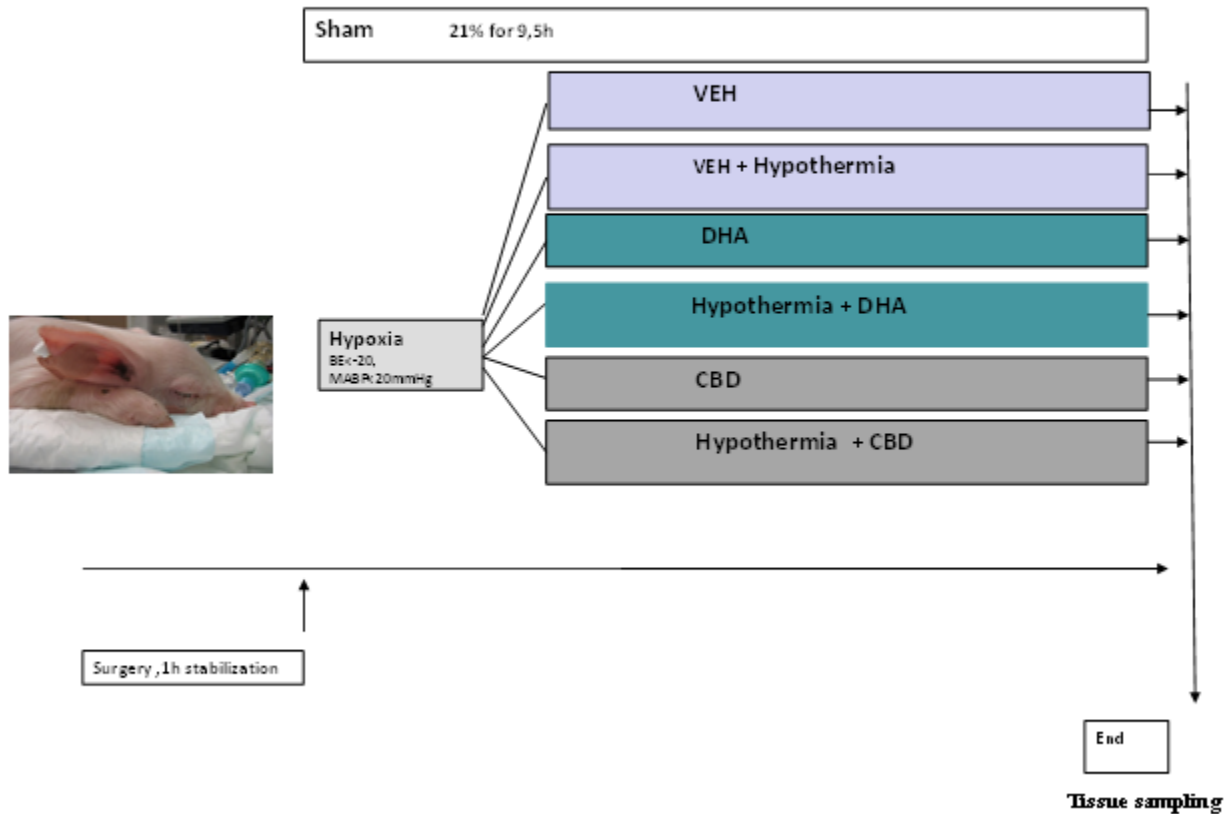

Supplement: S1 Fig — (PDF) [file pone.0201895.s001.pdf]
