# Supplementary material for: DHA and therapeutic hypothermia in a short-term follow-up piglet model of hypoxia-ischemia: Effects on H+MRS biomarkers
Source: PLoS One. 2018 Aug 7;13(8):e0201895. doi: 10.1371/journal.pone.0201895 (PMC6080779; doi:10.1371/journal.pone.0201895)
Supplement: S1 Table — Main effects of DHA and Hypothermia on H+MRS values shown as mean ppm ± SD. The two-way ANOVA gives the following groups: DHA (DHA pooled with DHA + HT), Non-DHA (VEH pooled with VEH + HT), HT (HT pooled with HT + DHA) and Non-HT (VEH + DHA). First column representing the interaction p-value between the main factors. Significant p-values in bold typing. * Interaction with p<0.10 with subsequent one-way ANOVA analysis and post-hoc Fisher’s test on the randomized goups (values in S2 Table). Non detectable (ND). (DOCX) [file pone.0201895.s002.docx]

**S1 Table. Main effects of H^+^MRS biomarkers.**

|  | **Interaction p-value** | **DHA** | | | **Hypothermia** | | |
| --- | --- | --- | --- | --- | --- | --- | --- |
| **Cortex** |  | DHA Non-DHA p-value | | | HT Non-HT p-value | | |
| NAA | 0.50 | 5.8 ± 0.6 | 6.9 ± 0.6 | 0.18 | 6.5 ± 0.6 | 6.3 ± 0.6 | 0.87 |
| Lac/NAA | 0.08 | 2.1 ± 0.3 | 2.5 ± 0.3 | * | 2.4 ± 1.9 | 2.2 ± 1.4 | * |
| Glu/NAA | 0.69 | 0.86 ± .04 | 0.85 ± .04 | 0.69 | 0.73 ± .2 | 0.95 ± .1 | **<0.0001** |
| GSH |  | ND | ND |  | ND | ND |  |
| **Hippocampus** |  |  |  |  |  |  |  |
| NAA | 0.10 | 6.0 ± 2.4 | 3.8 ± 1.5 | **0.001** | 4.7 ± 1.7 | 5.0 ± 2.7 | 0.36 |
| Lac/NAA | 0.94 | 2.1 ± .98 | 2.1 ± .57 | 0.83 | 1.8 ± .31 | 2.3 ± .49 | **0.003** |
| Glu/NAA | **0.03** | 0.88 ± .18 | 0.93 ± .32 | * | 0.73 ± .16 | 1.1 ± 0.21 | * |
| GSH | 0.22 | 0.41 ± .25 | 0.22 ± .17 | **0.009** | 0.29 ± .18 | 0.33 ± .27 | 0.41 |

Main effects of DHA and Hypothermia on H^+^MRS values shown as mean ppm ± SD. The two-way ANOVA gives the following groups: DHA (DHA pooled with DHA + HT), Non-DHA (VEH pooled with VEH + HT), HT (HT pooled with HT + DHA) and Non-HT (VEH + DHA). First column representing the interaction p-value between the main factors. Significant p-values in bold typing. * Interaction with p<0.10 with subsequent one-way ANOVA analysis and post-hoc Fisher’s test on the randomized goups (values in S2 Table). Non detectable (ND).
