# Supplementary material for: DHA and therapeutic hypothermia in a short-term follow-up piglet model of hypoxia-ischemia: Effects on H+MRS biomarkers
Source: PLoS One. 2018 Aug 7;13(8):e0201895. doi: 10.1371/journal.pone.0201895 (PMC6080779; doi:10.1371/journal.pone.0201895)
Supplement: S2 Table — (DOCX) [file pone.0201895.s003.docx]

**S2 Table. H^+^MRS biomarkers by randomized groups.**

|  | NAA | Lac/NAA | Glu/NAA | GSH |
| --- | --- | --- | --- | --- |
| Cortex |  |  |  |  |
| VEH | 7.2 ±2.6 | 1.9 ±0.5 | 1.0 ± 0.1 | ND |
| DHA | 5.4 ± 3.1 | 1.7 ±0.4 | 0.9 ± 0.2 | ND |
| VEH + HT | 6.7 ± 2.9 | 3.0 ±2.5 | 0.7 ± 0.3 | ND |
| DHA + HT | 6.1 ±3.1 | 1.7 ±0.4 | 0.7 ± 0.2 | ND |
| Hippocampus |  |  |  |  |
| VEH | 3.6 ± 1.8 | 2.5 ± 0.5 | 1.5 ± 0.2 | 0.21 ± 0.18 |
| DHA | 6.4 ± 2.9 | 2.5 ± 1.3 | 1.0 ± 0.2 | 0.49 ± 0.31 |
| VEH + HT | 4.0 ± 0.9 | 1.8 ± 0.4 | 0.7 ± 0.2 | 0.24 ± 0.18 |
| DHA + HT | 5.3 ± 2.0 | 1.9 ± 0.3 | 0.8 ± 0.1 | 0.34 ± 0.19 |
